# Increased apolipoprotein M induced by lack of scavenger receptor BI is not activated via HDL-mediated cholesterol uptake in hepatocytes

**DOI:** 10.1186/s12944-018-0849-7

**Published:** 2018-08-25

**Authors:** Yue-Hua Feng, Lu Zheng, Jiang Wei, Miao-Mei Yu, Jun Zhang, Guang-Hua Luo, Ning Xu

**Affiliations:** 1 Comprehensive Laboratory, the Third Affiliated Hospital, Soochow University, Changzhou, 213003 China; 2 Division of Clinical Chemistry and Pharmacology, Department of Laboratory Medicine, Lunds University, S-221 85 Lund, Sweden

**Keywords:** Apolipoprotein M, Scavenger receptor BI, Reverse cholesterol transport, Selective cholesterol uptake

## Abstract

**Background:**

Scavenger receptor BI (SR-BI) is a classic high-density lipoprotein (HDL) receptor, which mediates selective lipid uptake from HDL cholesterol esters (HDL-C). Apolipoprotein M (ApoM), as a component of HDL particles, could influence preβ-HDL formation and cholesterol efflux. The aim of this study was to determine whether SR-BI deficiency influenced the expression of ApoM.

**Methods:**

Blood samples and liver tissues were collected from SR-BI gene knockout mice, and serum lipid parameters, including total cholesterol (TC), triglyceride (TG), high and low-density lipoprotein cholesterol (HDL-C and LDL-C) and ApoM were measured. Hepatic ApoM and ApoAI mRNA levels were also determined. In addition, BLT-1, an inhibitor of SR-BI, was added to HepG2 cells cultured with cholesterol and HDL, under serum or serum-free conditions. The mRNA and protein expression levels of ApoM were detected by RT-PCR and western blot.

**Results:**

We found that increased serum ApoM protein levels corresponded with high hepatic ApoM mRNA levels in both male and female SR-BI^−/−^ mice. Besides, serum TC and HDL-C were also significantly increased. Treatment of HepG2 hepatoma cells with SR-BI specific inhibitor, BLT-1, could up-regulate ApoM expression in serum-containing medium but not in serum-free medium, even in the presence of HDL-C and cholesterol.

**Conclusions:**

Results suggested that SR-BI deficiency promoted ApoM expression, but the increased ApoM might be independent from HDL-mediated cholesterol uptake in hepatocytes.

## Background

Atherosclerosis is now one of the most common causes of death around the world, especially in advanced countries. High-density lipoprotein (HDL), which is always considered as a protective factor against atherosclerosis, serves as a vehicle for delivering excess cholesterol esters from peripheral tissues or cells to the liver and steroidgenic tissues. The process is called reverse cholesterol transport (RCT). Scavenger receptor class B type I (SR-BI) is a physiological high-affinity HDL receptor [1, 2]. It is most abundant in the liver, where it mediates selective cholesterol uptake from HDL-cholesterol (HDL-C) to hepatocytes in the later stage of RCT [3, 4]. Studies with mice showed that over-expression of SR-BI results in decreased HDL and total cholesterol (TC) concentrations, suppressing atherosclerosis by increasing RCT [5–9]. In contrast, down-regulation of hepatic SR-BI results in elevated HDL concentration, exaggerating atherosclerosis [10–12]. While in human, previous studies have identified that humans who carry mutations in a SCARB1 gene (a gene encoding human SR-BI), exhibited relatively higher plasma HDL-C levels and increased risk of cardiovascular disease [13, 14]. The above observations verified that a key role for hepatic SR-BI in both rodent and human cholesterol metabolism.

Apolipoprotein M (ApoM), an approximately 26 kDa protein, which is a constituent of plasma HDL [15], is mainly expressed in hepatocytes of the liver and tubular epithelial cells in kidney [16, 17]. Recently, many investigations have demonstrated that ApoM plays a critical role in lipid and lipoprotein metabolism. ApoM could delay oxidation of low-density lipoprotein cholesterol (LDL-C), influence preβ-HDL formation, hence mediating the antioxidant effect of HDL and acting as an acceptor of sphingosine-1-phosphate (S1P) [18–21]. It has been also suggested that ApoM is one of the important regulators of cholesterol metabolism and RCT, because ApoM-enriched HDL increases the ability of HDL to mobilize cellular cholesterol, and accelerates cholesterol efflux from macrophages in vivo [22]. Obviously, ApoM does promote the process of RCT. Since SR-BI is a key factor in RCT, this study was designed to investigate whether deficiency of SR-BI could affect the expression of ApoM, as well as its potential mechanisms.

## Methods

### Animals

SR-BI^−/−^ mice on a C57BL/6 background were kindly provided by Nanjing Medical University. The genotypes of the offspring of these mice were analyzed using the base-quenched probe method and gel electrophoresis, and all of them were housed in mesh stainless steel cages, maintained on a 12 h light-dark cycle with ad libitum access to a regular chow diet and tap water. The animals used in the experiments were 9–10 weeks old, and were picked out randomly. After 12 h fasting, blood samples for serum lipid and ApoM lipoprotein analysis were drawn into tubes with Na_2_EDTA and centrifuged for 10 min at 800×*g*. Serum samples were stored at − 80 °C until analysis. Livers were removed and immediately frozen after animals were sacrificed.

### Cell culture

HepG2 cells (American Type Culture Collection, Manassas, VA, USA) were maintained in DMEM containing 10% fetal calf serum. Cells were incubated at 37 °C in a humidified atmosphere containing 5% CO_2_. The HepG2 cells were seeded at a density of 6 × 10^5^/well on 6-well plates, and were grown to 70–80% confluence for 24 h before adding BLT-1 (Merck-Millipore, Darmstadt, Germany). Prior to the experiment, cells were washed twice with phosphate-buffered saline (PBS). Cells were cultured in the above medium or serum-free medium with 1% bovine serum albumin (BSA), containing one or more additives, i.e., BLT-1, HDL, and cholesterol, at different concentrations as described in the figure legends. HDL was purchased from Prospect Biosystems (Newark, NJ, USA), and cholesterol was obtained from Sigma-Aldrich (St. Louis, MO, USA).

### Genotypic analysis

Genomic DNA was isolated from mouse tail biopsies. Genotyping was performed by the base-quenched probe method [23], followed by melting curve analysis. The results were confirmed by gel electrophoresis [11]. Primers and probes specific for the targeted and wild-type SR-BI alleles are presented in Table 1.

**Table 1:** Specific Primers and Probe for Genotypic Analysis

Gene	Primer / Probe	Sequence (5′ to 3′)
SR-BI (Base-quenched Probe Technique)	Forward primer	AAGGAAGCCACGCCCACGCCTCACC
	Reverse primer 1	TCATGACAACGCCGAGCGCAGCAAAC
	Reverse primer 2	ATGCTGGGGATGCGGTGGGCTCTATG
	Probe	FAM-TCAGGTCCTGAGCGTCGAG-℗
SR-BI (Gel Electrophoresis)	Forward primer 2	AAGCCACGCCCACGCCTC
	Reverse primer 3	CCATCTCCCCAAGACACTTCACTCA
	Reverse primer 4	GATTGGGAAGACAATAGCAGGCATG

Note: FAM, 6-carboxyfluorescein; ℗, phosphate

### Total RNA extraction and real time RT-PCR

Total RNA from mouse liver tissues or HepG2 cells was isolated according to the manufacturer’s instructions using a total RNA purification kit (Shanghai Shenergy Biotech Co. Ltd., Shanghai, China). First-strand cDNA was synthesized from 2 μg of total RNA using the RevertAid™ first strand cDNA synthesis kit (Thermo Fisher Scientific, Waltham, MA, USA). Primer Premier 5.0 software (Premier Biosoft, Palo Alto, CA, USA) was used to design the mouse and human ApoM primers and probe used in the TaqMan assay, shown in Table 2. Relative standard curves for ApoM, ApoAI, and GAPDH were generated to compensate for the efficiency of PCR. ApoM and ApoAI mRNA levels were quantified and expressed relative to GAPDH mRNA levels, and were performed on a LightCycler480®II in a final volume of 25 μL. Optimum reaction conditions were obtained with 0.1 μL of 100 μM of each primer and probe, 2.5 μL of 10× PCR buffer, 1.5 μL of 25 mM MgCl_2_, 0.5 μL of 10 mM 4× dNTPs, 0.25 μL of 5 U/μL Taq DNA polymerase, and 2 μL template cDNA. Finally 17.95 μL ddH_2_O was added to the reaction mixture. The mixture was preheated at 95 °C for 1 min to activate Taq polymerase, followed by 40 cycles at 95 °C for 5 s and 60 °C for 15 s. Samples were amplified simultaneously in duplicate in one assay run. The threshold cycle (CT) was defined as the fractional cycle number at which the reporter fluorescence reached a certain level.

**Table 2:** Sequences of primers and probes for real-time RT-PCR

Gene	Primer / Probe	Sequence (5’ to 3’)
Mouse ApoM	Forward	GCTTTCTCCTCTACAATCGGTCAC
	Reverse	CGGGCAGGCCTCTTGATT
	Probe	FAM- ACCTCTTGCTTGGACTTCAAAGCCTTCTTA-TAMRA
Mouse ApoAI	Forward	CAGTTTGAATCCTCCTCCTTGG
	Reverse	GGTTATCCCAGAAGTCCCGAG
	Probe	FAM-CAACAGCTGAACCTGAATCTCCTGGAA-TAMRA
Mouse GAPDH	Forward	TCTTGTGCAGTGCCAGCCT
	Reverse	TGAGGTCAATGAAGGGGTCG
	Probe	FAM-AGGTCGGTGTGAACGGATTTGGC-TAMRA
Human ApoM	Forward	CTGACAACTCTGGGCGTGGAT
	Reverse	TGTCCACAGGGTCAAAAGTTGC
	Probe	FAM-AGTTCCCAGAGGTCCACTTGGGCCA-BHQ1
Human GAPDH	Forward	CAGGGCTGCTTTTAACTCTGGT
	Reverse	CATGGGTGGAATCATATTGGAAC
	Probe	CY5-TGGATATTGTTGCCATCAATGACCCCT-BHQ2

### Western blot analysis

ApoM concentrations in serum and HepG2 cells were determined by western blot analyses [24]. In brief, serum was diluted in PBS (1:50), then 20 μL diluted samples or 20 μg protein extracted from HepG2 cells were separated by 12% SDS-polyacrylamide gel electrophoresis (SDS-PAGE). After transferring onto polyvinylidene difluoride (PVDF) membranes, the blots were incubated at 37 °C with anti-ApoM or anti-GAPDH antibodies for 2 h, then with horseradish peroxidase-conjugated goat anti-rabbit IgG for 1 h. Bands were visualized by an ECL + Plus western blotting detection system (CW Biotech, Beijing, China) and quantified using a scanner with Quantity One software (Version 4.2.1, Bio-Rad Laboratories, Hercules, CA, USA).

### Biochemical analysis

Serum TC, triglyceride (TG), HDL-C and LDL-C were detected by standard clinical chemical methods.

### Statistical analysis

Data are expressed as the mean ± SD. Statistical analyses were conducted using GraphPad Prism (version 5.0). Comparisons among groups were analyzed by one-way ANOVA followed by unpaired Student’s *t*-test. A *p* value < 0.05 was considered statistically significant.

## Results

### Genotyping results of SR-BI gene knockout mice

**Fig. 1:**
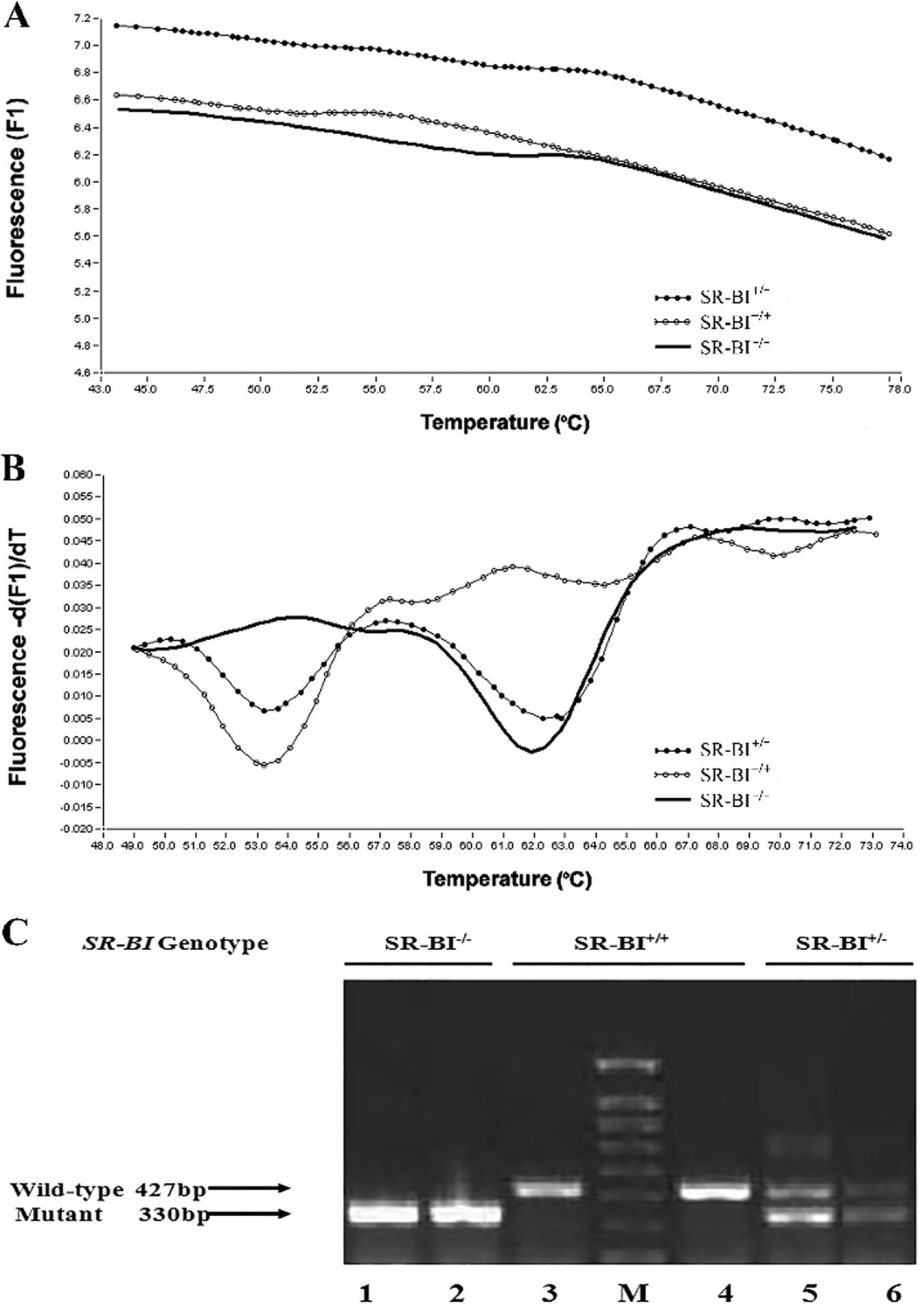
PCR-based genotyping analysis, confirmed by gel electrophoresis. **a** Curves of fluorescence (F) versus temperature (T) for sequence-specific base-quenched probe complementary to the SR-BI knockout gene sequence. **b** Derivative melting curves (-dF/dT vs. T) that depict the same data shown in panel A, wild-type (SR-BI^+/+^), heterozygous (SR-BI^+/−^), and homozygous (SR-BI^−/−^) mutant mice. **c** Two sets of primer pairs specific for the wild-type (primers 2 and 3) or targeted mutant (primers 2 and 4) alleles were used to screen genomic DNA by PCR as described. Representative results from wild-type (SR-BI^+/+^, lanes 3 and 4), heterozygous (SR-BI^+/−^, lanes 5 and 6), and homozygous mutant (SR-BI^−/−^, lanes 1 and 2) animals are shown

The SR-BI^+/−^ and SR-BI^−/−^ mice looked normal (weight, general appearance, and behavior). Therefore, we designed primers and probes to amplify the targeted segment by two means: the base-quenched probe method and gel electrophoresis. According to the base-quenched probe technique, the mutant allele achieved a perfect match resulting in a high melting temperature of 62.25 ± 0.31 °C, compared with the WT allele (53.25 ± 0.81 °C), while the SR-BI^+/−^ mice showed two melting valleys (Fig. 1a and b). For gel electrophoresis, the amplification of a 330 bp fragment represented the deletion of SR-BI allele, whereas a fragment of 427 bp represented the WT allele, and the presence of both fragments suggested heterozygous mice (Fig. 1c). The results of over 200 mice showed consistency between the two methods.

### Serum lipid concentrations in mice

As shown in Table 3, serum TC levels in the SR-BI^+/−^ group were significantly increased relative to WT controls, (both males and females, *p* < 0.01) and extremely higher in the SR-BI^−/−^ group (both males and females, *P* < 0.001); HDL-C concentrations were also raised in the SR-BI^−/−^ group (males, *P* < 0.05 and females, *P* < 0.001); but there was no significant difference in the serum TG levels or in LDL-C concentrations among the different genotypes. However, the SR-BI^−/−^ group showed an obviously abnormal lipid metabolism.

**Table 3:** Serum lipid parameters in each group

*SR-BI* genotype	Gender	N	TC (mmol/L)	TG (mmol/L)	HDL-C (mmol/L)	LDL-C (mmol/L)
SR-BI^+/+^	Male	6	1.84 ± 0.19	0.88 ± 0.17	1.22 ± 0.11	0.15 ± 0.04
	Female	6	1.77 ± 0.17	0.76 ± 0.11	1.05 ± 0.06	0.18 ± 0.03
	Both	12	1.81 ± 0.18	0.82 ± 0.15	1.13 ± 0.12	0.16 ± 0.03
SR-BI^+/−^	Male	6	2.40 ± 0.40	1.14 ± 0.37	1.46 ± 0.22	0.19 ± 0.04
	Female	5	2.20 ± 0.22^*c*^	0.78 ± 0.17	1.21 ± 0.08^*c*^	0.20 ± 0.05
	Both	11	2.31 ± 0.33^*e*^	0.98 ± 0.34	1.35 ± 0.21	0.20 ± 0.04
SR-BI^−/−^	Male	5	4.26 ± 0.64*^a^	0.94 ± 0.16	1.71 ± 0.46*	0.21 ± 0.10
	Female	5	4.11 ± 0.40^*bd*^	0.86 ± 0.16	1.53 ± 0.10^*bd*^	0.18 ± 0.04
	Both	10	4.19 ± 0.51^*fg*^	0.90 ± 0.16	1.62 ± 0.33^*fh*^	0.21 ± 0.08

Note: TC (total cholesterol), TG (Triglycerides), HDL-C (high density lipoprotein cholesterol), LDL-C (low density lipoprotein cholesterol), values are representative of both sexes, *n* = 5–6 per group, mean ± SD

1) Males:*P < 0.05 vs. SR-BI^+/+^ group; ^*a*^*P* < 0.01 vs. SR-BI^+/−^ group;

2) Female:^*b*^*P* < 0.001 vs. SR-BI^+/+^ group; ^*c*^*P* < 0.01 vs. SR-BI^+/+^ group; ^*d*^*P* < 0.001 vs. SR-BI^+/−^ group;

3) Both:^*e*^*P* < 0.01 vs. SR-BI^+/+^ group; ^*f*^*P* < 0.001 vs. SR-BI^+/+^ group; ^*g*^*P* < 0.001 vs. SR-BI^+/−^ group; ^*h*^*P* < 0.01 vs. SR-BI^+/−^ group

### Serum ApoM protein and hepatic ApoM mRNA levels in the different groups

**Fig. 2:**
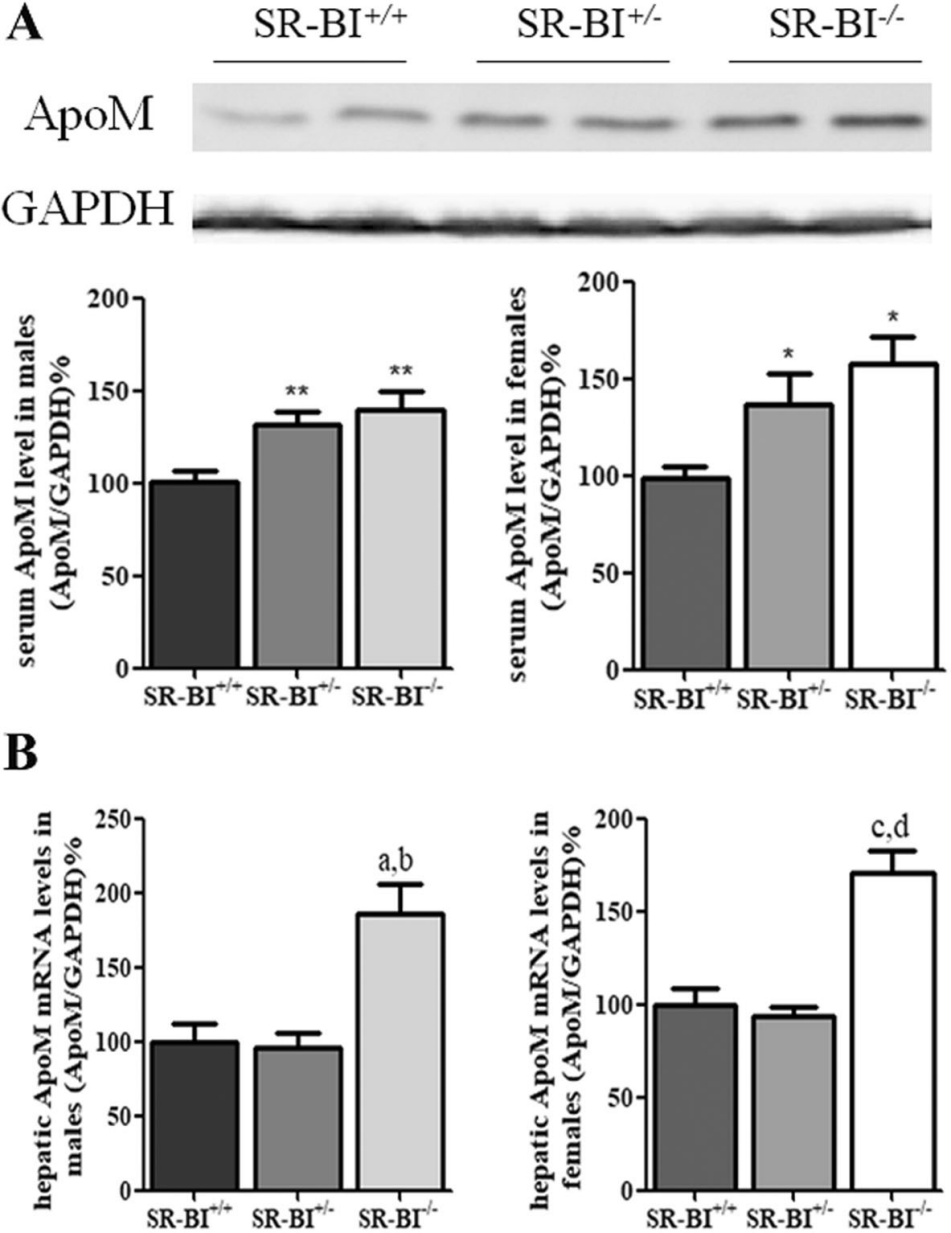
Serum ApoM and hepatic ApoM mRNA levels in mice. **a** Western blot analysis of serum ApoM levels in different groups of SR-BI mice. The SR-BI^+/+^ males or SR-BI^+/+^ females were set at 100% as respective controls. **b** Hepatic ApoM mRNA levels in different groups of SR-BI mice. The SR-BI^+/+^ males or SR-BI^+/+^ females were set at 100% as respective controls. *n* = 5–6 per group, mean ± SD, ^**^P < 0.01 vs. SR-BI^+/+^ group; ^*^P < 0.05 vs. SR-BI^+/+^ group; ^a^*P* < 0.001 vs. SR-BI^+/+^ group; ^b^*P* < 0.01 vs. SR-BI^+/−^ group; ^c^*P* < 0.001 vs. SR-BI^+/+^ group; ^d^*P* < 0.001 vs. SR-BI^+/+^ group

To investigate whether knockout of SR-BI affected the expression of ApoM in vivo, we detected the protein ApoM in the serum of the different groups. Serum ApoM protein levels were significantly higher in the SR-BI^−/−^ group compared to WT controls (*P* < 0.01) and the SR-BI^+/−^ group (*P* < 0.05), but there was no significant difference between the SR-BI^+/−^ group and WT controls (Fig. 2a). However, serum ApoAI protein concentrations showed no differences among them (data not shown). Consistent with this, hepatic ApoM mRNA levels were much higher in the SR-BI^−/−^ group than the others. In the livers of SR-BI^−/−^ mice, ApoM levels increased by 186% and 170% in males and females relative to WT controls, respectively (*P* < 0.001) (Fig. 2b). Similarly to serum, there were no obvious differences in hepatic ApoAI mRNA levels among the groups (data not shown).

### Effects of BLT-1 on the expression of ApoM in HepG2 cells

**Fig. 3:**
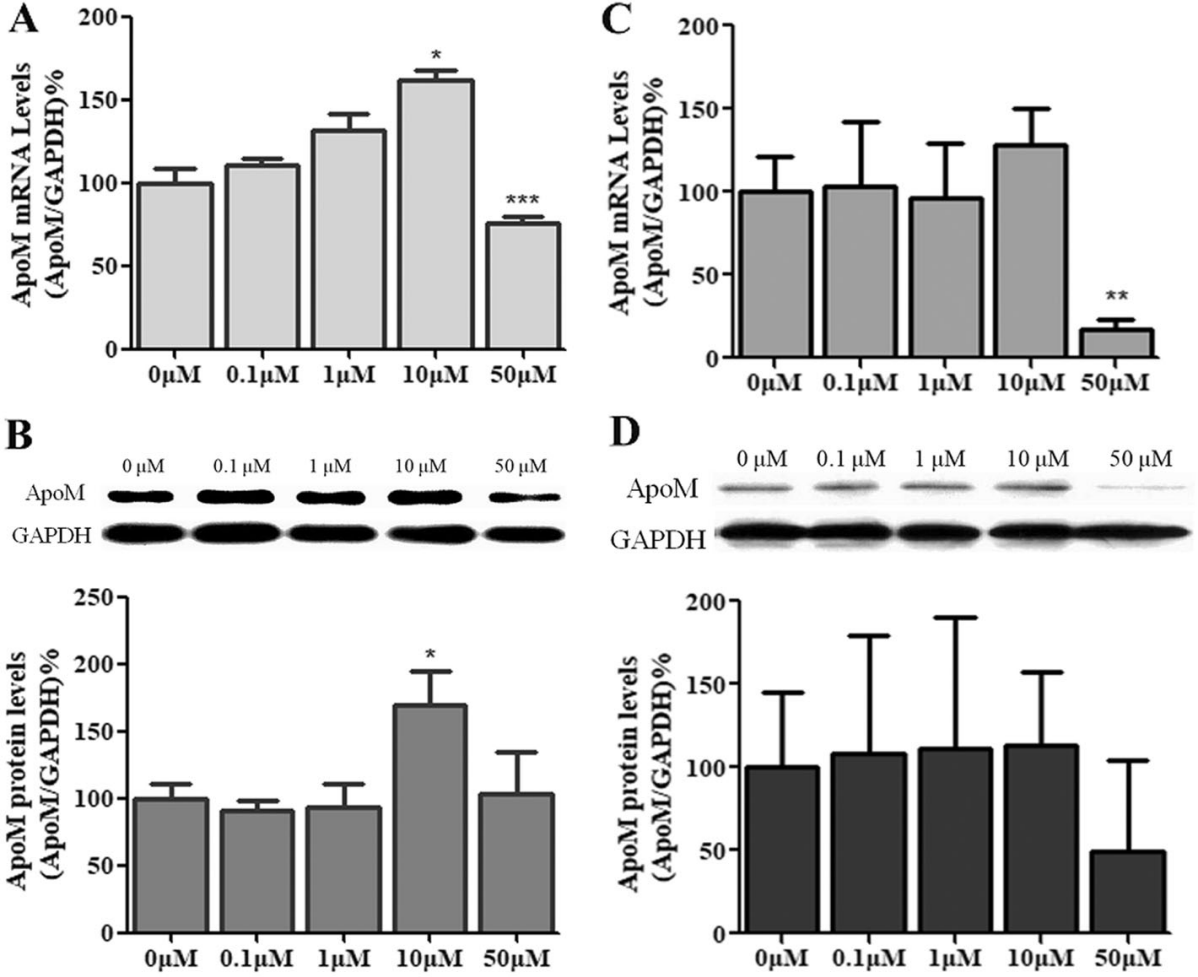
Effects of BLT-1 treatment on ApoM expression in serum-containing medium or serum-free medium. HepG2 cells were treated with 0, 0.1, 1, 10, or 50 μM BLT-1 in serum-containing medium (**a** and **b**) or serum-free medium with 1% BSA (**c** and **d**) for 12 h. ApoM mRNA levels were determined by RT-PCR (**a** and **c**). ApoM protein levels were measured by western blotting (**b** and **d**). Each group contained six replicates, and data are presented as the mean ± SD. The 0 μM group was set at 100% as control. ^*^P < 0.05 vs. 0 μM group; ^**^P < 0.01 vs 0 μM group; ^***^P < 0.001 vs 0 μM group

To further validate the effects of SR-BI deficiency on ApoM expression, we treated HepG2 cells with BLT-1, a specific inhibitor of SR-BI. When cells were incubated with 10% fetal calf serum, the mRNA levels of ApoM increased in HepG2 cells treated with 10 μM BLT-1 (*P* < 0.05) compared to controls (Fig. 3a). Consistent with this, ApoM protein levels were significantly elevated (*P* < 0.05) (Fig. 3b). When cells were cultured in serum-free medium with 1% BSA, there was no significant change in the expression of ApoM, either in mRNA or in protein levels (Fig. 3c and d).

### Effects of BLT-1, HDL and cholesterol on the expression of ApoM in HepG2 cells

**Fig. 4:**
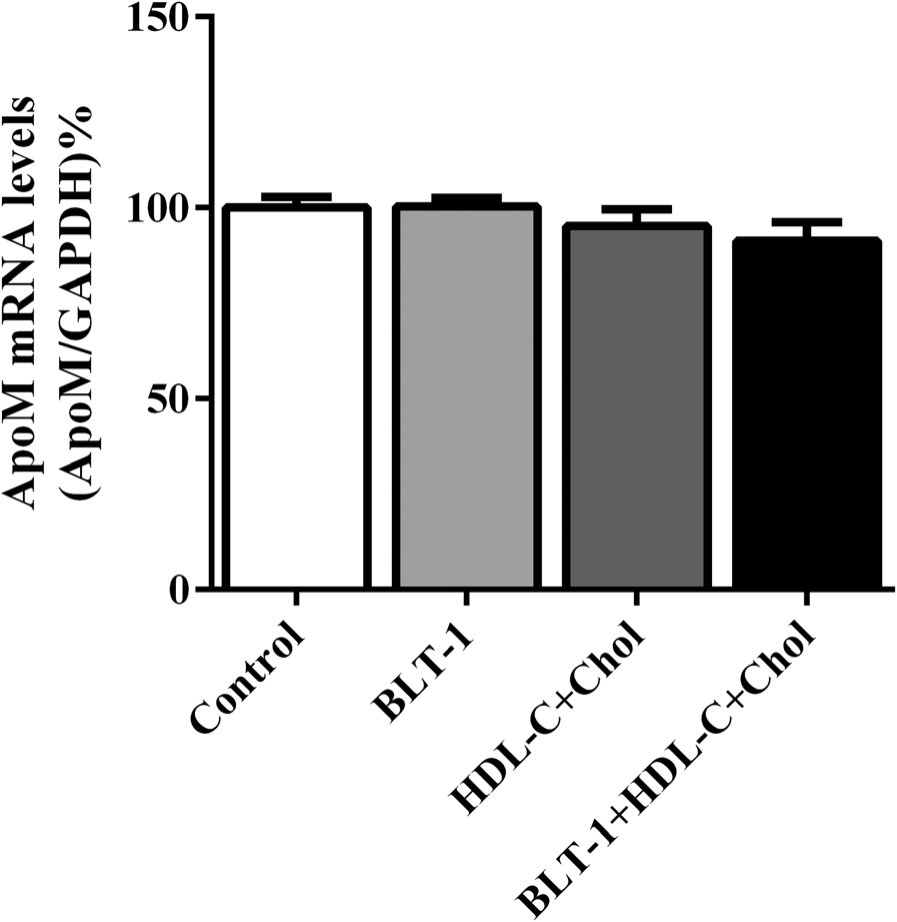
Effects of HDL-C, cholesterol, and BLT-1 on ApoM expression in HepG2 cells. ApoM mRNA levels in cells cultured with HDL-C, cholesterol and BLT-1 for 12 h were measured by RT-PCR. The control group without HDL-C, cholesterol or BLT-1 was set as 100%. *n* = 6 for each group, data are presented as the mean ± SD

As shown in Fig. 4, there was no difference in mRNA levels of ApoM among groups including cells treated with BLT-1 alone, with HDL-C and cholesterol, and with HDL-C, cholesterol together with BLT-1.

## Discussion

The aim of the present study was to explore whether lack of SR-BI could affect the expression of ApoM. Our results indicated that the absence of SR-BI did up-regulate ApoM expression, both in mRNA and protein levels in SR-BI gene knockout mice (Fig. 2), which suggested SR-BI could regulate expression of ApoM. To investigate the potential mechanism, we used BLT-1, a specific inhibitor of SR-BI, to treat HepG2 hepatoma cells. The results showed the mRNA levels of ApoM increased significantly in HepG2 cells treated with 10 μM BLT-1 in presence of 10% fetal calf serum, but not in absence of serum (Fig. 3a). These results strongly suggested that BLT-1 may prevent some serum factors not yet identified transferring into cells, which could influence the expression of ApoM. It should be point out that the high concentrations of BLT-1 induced cell death, for cells treated with 50 μM BLT-1 were mostly detached (Fig. 3). To further validate whether the regulation of ApoM is due to RCT induced by SR-BI in hepatocytes, we treated HepG2 cells with HDL-C, cholesterol and BLT-1 in serum-free medium with 1% BSA, respectively. The results showed that ApoM expression was not affected by HDL-C and cholesterol, which indicated that cholesterol metabolism might not regulate ApoM expression. Even though BLT-1 was used to inhibit the function of SR-BI, ApoM expression was not changed significantly (Fig. 4). It suggested SR-BI-mediated selective cholesterol uptake did not involve in regulation of ApoM.

In the present study, we found lack of SR-BI caused TC and HDL-C accumulation, which was consistent with previous studies [11]. As s constituent of HDL, serum ApoM level also increased in SR-BI deficient mice. We consider that this phenomenon may due to the absence of SR-BI, which causes negative feedback and leads to up-regulation of HDL-C, including ApoM. Further investigation demonstrated that inhibition of SR-BI by BLT-1 also elevated ApoM expression in HepG2 cells treated with medium containing fetal calf serum. Interestingly, the phenomenon was not observed in medium without serum. These results implied there may be some feedback regulators existing in serum. In conclusion, deficiency of SR-BI could up-regulate ApoM, which may be due to negative feedback rather than HDL-mediated cholesterol uptake in hepatocytes. The detailed mechanism remains to be elucidated.

## Abbreviations

ApoM: Apolipoprotein M
DMEM: Dulbecco’s Modified Eagle’s Medium
FBS: Fetal bovine serum
GAPDH: Glyceraldehyde-3-phosphate dehydrogenase
HDL-C: High density lipoprotein-cholesterol
LDL-C: Low density lipoprotein-cholesterol
PBS: Phosphate buffered saline
PCR: Polymerase chain reaction
TC: Total cholesterol
TG: Triglyceride
